# Challenges of women refugees in utilising reproductive health services in public health institutions in Durban, KwaZulu-Natal, South Africa

**DOI:** 10.4102/hsag.v24i0.1030

**Published:** 2019-08-14

**Authors:** Yvonne Munyaneza, Euphemia M. Mhlongo

**Affiliations:** 1 School of Nursing and Public Health, University of KwaZulu-Natal, Durban, South Africa

**Keywords:** public healthcare facilities, refugee, reproductive health, women, challenges

## Abstract

**Background:**

Reproductive health services are essential for everyone worldwide. In South Africa, the available literature does not address reproductive health as a full package for women refugees and their experiences. This study addressed women refugees in relation to reproductive healthcare services they receive from public healthcare facilities.

**Aim:**

The aim of the study was to document the day-to-day experiences of women refugees and uncover their challenges regarding utilisation of reproductive health services in public institutions of Durban, KwaZulu-Natal.

**Setting:**

The study was conducted in eThekwini district, Durban, KwaZulu-Natal, and did not consider participants who are located beyond the above-mentioned city’s borders.

**Methods:**

A qualitative, descriptive design was used. A semi-structured interview guide was used to collect data through face-to-face in-depth interviews with eight women refugees. Thematic content analysis guided the study.

**Results:**

Two major themes emerged: negative experiences or challenges, and positive experiences. The most dominant negative experiences included medical xenophobia and discrimination, language barrier, unprofessionalism, failure to obtain consent and lack of confidentiality, ill-treatment, financial challenges, internalised fear, religious and cultural hegemony, and the shortage of health personnel and overcrowding of public hospitals. The positive experiences included positive treatment and care and social support.

**Conclusion:**

The findings revealed that women refugees in Durban, KwaZulu-Natal, face many challenges such as medical xenophobia and discrimination in their attempt to seek reproductive health services in public healthcare facilities, making them even more vulnerable. Assisting women refugees with their reproductive health needs will remediate the challenges they face.

## Introduction

It is documented that South Africa hosts more refugees than the rest of the sub-region. Of the 449 000 refugees in the sub-region, South Africa alone hosts 65 226 refugees, excluding the undocumented and uncounted asylum seekers (Zihindula et al. 2016). According to the United Nations High Commission for Refugee (UNHCR), there are approximately 46.2 million refugees worldwide and most of them are war related (UNHCR 2015). In addition to facing challenges related to documentation, language barrier and negative attitudes from the citizens in the host countries, most refugees are also left unprotected and exposed to human rights violations (Mujawamariya 2013). Sub-Saharan Africa is the most affected region, and specifically the central African countries, namely Burundi, Rwanda and the Democratic Republic of Congo (DRC). This region has been unstable since 1994 because of the war and conflicts that started in Rwanda and later affected its neighbours, and the people have been on a run since then. While many of those who flee these countries have sought refuge in Europe and America, studies have shown that a considerable number have landed in the Southern African region, specifically in South Africa (Landau 2014; Vearey 2014). It was indicated by Zihindula, Meyer-Weitz and Akintola (2015a) that the most affected refugee group is women because of their specific needs for reproductive health services.

According to the 1951 convention on refugee status, a refugee is defined as someone who is unable or unwilling to return to their country of origin owing to a well-founded fear of being persecuted for reasons of race, religion, nationality, membership of a particular social group or political opinion (Phillips 2013).

For the purposes of this study, the definition by the International Conference on Population and Development (ICPD 1994) was adopted, as it describes reproductive healthcare in the context of primary healthcare to include family planning; antenatal care, safe delivery and post-natal care; prevention and appropriate treatment of infertility; prevention of abortion and management of its consequences; treatment of reproductive tract infections; care and treatment of sexually transmitted infections (STIs) and HIV and AIDS; information, education and counselling on reproductive health; prevention and surveillance of violence against women; care for survivors of violence and other actions to eliminate traditional harmful practices; and appropriate referrals for further diagnosis and management of the above.

Upon their arrival in the host country, women are at high risk of contracting different types of illnesses (Apalata et al. 2007) most of which are related to their reproductive health. Even in the host country, women refugees are still in need of reproductive health services such as family planning, safe delivery, antenatal and post-natal care, and care and treatment of STIs. They also need information, education and counselling on reproductive health. During the literature search for this study, no study was found that documents the experiences of these women refugees with regard to reproductive health services in South Africa. This lack of documentation is a threat not only to the refugee community, but also to the host because after integration in the host community, there is inter-marriage and cohabitation between local people and refugees. While during the literature search, no study was found that focuses on and documents the experiences of women refugees with regard to reproductive health services in South Africa, the existing limited information looks at access to healthcare services at large (Crush & Tawodzera 2014; Nkosi 2014; Vearey 2014).

However, there are international studies that attempted to explore the experiences of women refugees and migrants and their reproductive health needs. In the study conducted in Geneva, the researchers discovered that migrant women experience more complicated pregnancy outcomes and fewer migrant women access preventive gynaecology services because of a number of barriers such as financial accessibility, language barrier, discrimination and lack of information (Schmidt et al. 2018). Women refugees were classified as a vulnerable group with regard to pregnancy outcomes and accessing maternal care (Asif, Baugh & Jones 2015). The current study will bridge that gap with empirical data that will be generated from in-depth interviews with women refugees attending public health facilities in Durban, KwaZulu-Natal, South Africa. Finally, this work will contribute to policy changes and implementation, and health system improvements, which will ultimately enhance the quality of life of women refugees living in South Africa.

This research targeted women refugees who refer to themselves as refugees regardless of the possession or non-possession of refugee permits or refugee status. This study has, therefore, prioritised women refugees’ health and well-being with a particular focus on their reproductive health and the challenges they face in utilising reproductive health services in public health institutions.

The purpose of this qualitative study was to document the day-to-day experiences of women refugees and uncover their challenges regarding utilisation of reproductive health services in public health services in Durban, KwaZulu-Natal.

### Problem statement

The number of women refugees in South Africa continues to grow as the number of refugees and foreigners entering South Africa is increasing (Dalton-Greyling & Campus 2008). Among them are women from the Great Lakes region, who because of the war situation in their countries have been exposed to health risks from their home, along the way to their destination, and carried those risks into the host country. Upon their arrival in South Africa, the women refugees do not undergo any screening, yet many of them are survivors of rape and many other acts of sexual violence. There is paucity of literature on the experiences of women refugees regarding access to reproductive health services in South Africa. This lack of documentation is a threat not only to the refugee community, but also to the host because after integration in the host community, there are inter-marriages and cohabitation between local people and refugees. Furthermore, the latter are continuously reproducing despite the socio-economic life conditions that they are living under.

### Research methods

This study was founded on the interpretivist paradigm that operates on the premise that knowledge and the way it is studied are dynamic, contextual and may be dependent on the perspectives of different participants, be they researchers, policy-makers or other consumers of such knowledge (Kiura 2012).

## Research design

A qualitative descriptive design was adopted. The design provided an opportunity for ‘thick and rich’ descriptions of the participants’ experiences and challenges they face in utilising reproductive healthcare services, and offered a chance to individuals whose voices are rarely heard (Ulin et al. 2002).

### Population

This study was conducted in KwaZulu-Natal, South Africa among women refugees from different countries. The study population included women refugees who live in the City of Durban, KwaZulu-Natal, aged between 18 and 49 years and have sought reproductive health services at one of the public institutions.

### Sampling

A purposive sampling method was used because the researcher wanted to seek out and sample only women refugees who live in the City of Durban, KwaZulu-Natal, and have sought reproductive health services in any public hospitals or clinics in the said region. Purposive sampling allowed the researcher to select participants who are able to provide rich information about the phenomenon that is being studied (Creswell 2009). A total of 10 participants were purposively sampled among women refugees provided that they were aged between 18 and 49 years, and have sought reproductive health services at one of the public institutions; they are nationals from either one of the three Great Lakes countries.

### Data collection methods

Qualitative data for this study were collected using face-to-face in-depth interviews with eight participants and data saturation was reached. The data collection tool for the study was developed by the researcher and included questions related to the study’s aims and objectives. The main guiding question of the interview was: ‘what are the day-to-day experiences of women refugees regarding utilisation of the reproductive health services in Durban’s public healthcare facilities?’. The interviews were audio-recorded using the researcher’s cell phone and later transcribed to capture the interviewees’ answers in their own terms and also allow thorough examination of what participants said. In addition to audio-recording, the researcher took notes during the interviews which she later transcribed. The interviews were held at the Divine Mission Church, and Paran Pentecostal Church venues were usually used for meetings and functions at suitable times for each participant. The interviews were in English, and clarification and reformulation of the questions were done by the researcher where it was needed to assist the participants with a better understanding. The interviews took from 30 to 45 min except for the one that lasted 26 min. Data were transcribed on completion of each interview.

### Data analysis

Data were analysed following thematic analysis. Data analysis was done after each interview to allow the researcher to monitor data saturation. The in-depth interviews were transcribed verbatim. The researcher started by transcribing the recorded data at the same time paying attention to the notes taken during each interview with the participants, to ensure no information is left behind. Then the researcher read and re-read the transcribed data, highlighting, identifying and organising the main categories with similar ideas, which she later grouped into themes and sub-themes to help her outline the analysis before the actual analysis took place. The themes and sub-themes were checked and approved by the supervisor.

### Trustworthiness

Criteria, as set out by Lincoln and Guba (1985), were used to establish the trustworthiness of qualitative data. Notes were written during face-to-face interviews and transcribed immediately after each interview to ensure credibility of the study. The interviews were audio-recorded, and then transcribed verbatim to make sure that all participants’ responses are captured appropriately. Following the transcription of the recorded interviews, all participants were given a chance to review the transcribed interviews and were asked to confirm the accuracy of information they gave during data collection. To ensure transferability, there was rich and thorough description of the research process and the research setting.

### Ethical considerations

The study protocol was approved by the Ethics Committee of the University, ethical clearance number: HSS/0224/016M. Permission was also obtained from the church leaders who are the senior Pastors of Durban Mission and Paran Pentecostal Ministries churches as the study involved their church members. Participants were informed about their right to withdraw from the study at any time should they wish to do so, and were given an information letter on the study, and they willingly signed an informed consent. Pseudonyms were used to protect the identity of the participants. The ethical principles as described (Emanuel et al. 2004) were taken into account, and referral mechanisms and psychological support measures were also put in place to deal with discomfort or distress because of discussing the negative experience. The measures included referral to a psychologist in the nearest hospital, referral to their church prayer support where the pastor counsels and prays for members who experience stressful events.

## Findings

### Demographic characteristics of participants

This study intended to sample 10 participants from the three Great Lakes countries: Rwanda, Burundi and DRC. However, as the sample size was guided by data saturation, a total of eight women refugees were interviewed using a semi-structured interview guide. The participants’ ages ranged between 24 and 48 years old, two of them were from Burundi, four from Rwanda and two were DRC nationals. Of these, six were married and two were single. A total of five had a diploma from their home countries, one studied up to the second year of secondary school and two had matric; one of the latter was still studying in the university. Table 1 presents the demographic data for all the participants who were included in the study.

**TABLE 1:** Demographic characteristics of participants.

Pseudonym	Age	Level of education	Country of origin	No. of children	Marital status	Type of document	Occupation
Maria	43	Diploma	Rwanda	3	Married	Refugee status	Unemployed
Amanda	48	Diploma in teaching	Rwanda	4	Married	Refugee status	Social worker
Patricia	25	Matric	Burundi	2	Single	Refugee status	Unemployed
Nadine	40	2 years of secondary school	Burundi	2	Married	Refugee status	Small business
Mercy	36	Diploma	Rwanda	3	Married	Refugee status	School librarian assistant
Daniela	26	Diploma	DRC	1	Married	Refugee status	Housewife
Martha	24	Matric	Rwanda	None	Single	Refugee status	Student
Princess	47	Diploma	DRC	3	Married	Refugee status	Housewife

DRC, Democratic Republic of Congo.

### Experiences of women refugees in utilising reproductive health services in public health institutions

Two major themes emerged from the interviews that included negative experiences or challenges and positive experiences. The sub-themes for negative and positive experiences are displayed in Table 2.

**TABLE 2:** Themes and sub-themes.

Themes	Sub-themes
1. Negative experiences or challenges	1.1. Medical xenophobia 1.2. Discrimination 1.3. Language barrier 1.4. Unprofessionalism 1.4.1. Lack of health education and customer care training 1.4.2. Failure to obtain consent and lack of confidentiality 1.4.3. Ill-treatment 1.5. Internalised fear 1.6. Financial challenges 1.7. Shortage of health personnel and overcrowding of public hospitals 1.8. Religious and cultural hegemony
2. Positive experiences	2.1. Positive treatment and care 2.2. Social support

### Theme 1: Negative experiences or challenges

#### Sub-theme 1.1 and 1.2: Medical xenophobia and discrimination

Xenophobia is the most serious barrier to healthcare access for refugees. In this study, the findings revealed that all participants experienced xenophobia and discrimination in public healthcare facilities while seeking reproductive health services. They reported facing negative attitudes from healthcare professionals, mostly nurses, because of the fact that they are non-citizens. The study also revealed that in some instances the participants were neglected and others denied treatment because they were foreigners. This was evidenced by the following statements made by the participants:

‘She stood up from her chair shouting and pointing fingers at me saying “you foreigners you are using our hospitals but you don’t pay tax and you like to have babies”. I didn’t know what to say so I walked out of the room. I was afraid so I left that clinic and never went back.’ (Nadine, 40 years old, married)

‘The day I went to give birth it was the worst day of my life, there was no one to take care of me. I was calling the nurse telling them that I feel like pushing and I was feeling the baby’s head coming out but no one came. When I called the nurse who was around she said “You kwerekwere you always like to scream just keep quiet you are still far…”’ (Daniela, 26 years old, married)

#### Sub-theme 1.3: Language barrier

Language barrier may lead to medical errors by impending patient-healthcare provider communication. The findings revealed that women refugees face challenges of language barriers and bad attitudes from the healthcare workers in public institutions when seeking reproductive health services. It was pointed out that the majority of nurses insist on speaking isiZulu to the foreigners who hardly understand a word of it. It was also noted that while attending the public sector for reproductive health services, the women who are able to speak isiZulu are helped efficiently, as opposed to women refugees and other foreign nationals who cannot explain themselves in isiZulu. The findings indicated that even when the women refugees try to communicate in English, the nurses do not pay attention simply because they want foreigners to speak in isiZulu. Participants also reported that they have no interpreters and that they struggle to communicate with the healthcare professionals. This is evidenced by the following statements:

‘It was quite difficult, I couldn’t communicate. I used to take someone along with me to help me translate. It was still difficult because the translator was also a refugee and her English wasn’t good but she was better than me. Even so the nurses insisted in talking in Zulu and I didn’t understand a word of it.’ (Patricia, 25 years old, single)

‘We were so many in the clinic and the nurse spoke to me in Zulu; when I tried to tell her that I did not understand asking her to explain in English and she shouted saying, “You people go back to your country we are tired of you.”’ (Daniela, 26 years old, married)

#### Sub-theme 1.4: Unprofessionalism

Unprofessionalism is defined as not conforming with the standards of a profession or unprofessional behaviour (Campbell & Taylor 2008). All the participants encountered unprofessionalism from the nurses in the public hospitals and clinics. This sub-theme includes lack of health education and lack of good customer care training, failure to obtain consent and lack of confidentiality, and ill-treatment

**Subtheme 1.4.1: Lack of health education and customer care training:** Lack of health education and lack of good customer care training were identified as examples of unprofessionalism on the part of healthcare workers. Lack of understanding of the refugees’ situation and their rights to healthcare services and limited or non-existent good customer care behaviour affect refugees’ healthcare access and usage. The findings revealed that nurses in the public sector lack training in how to treat or deal with vulnerable people, in this case women refugees in the host country. The participants put it this way:

‘So, they want to force us to take injections or tablets without even explaining to us which one is better.’ (Maria, 43 years old, married)

‘I went to the hospital for family planning and the first thing a nurse wanted to do was to inject me and I quickly refused. I wanted tablets and she just gave me without explaining anything so I had to ask people outside.’ (Amanda, 48 years old, married)

**Subtheme 1.4.2: Failure to obtain consent and lack of confidentiality:** The findings revealed that the healthcare providers fail to get consent from clients before doing medical procedures and giving medication. Lack of confidentiality mainly because of the fact that the women refugees are obliged to have a third party to translate into local languages when interacting with healthcare providers was also highlighted. This is noted in the quotations below:

‘The c/section wasn’t a good experience in government hospital, it was something forced onto me and I was tricked to signing consent but they did not explain that I was going to have an operation. Then later I was forced to take injection without even advising me about it.’ (Patricia, 25 years old, single)

‘There are things a translator is not supposed to know; with a language barrier your problems are not confidential anymore.’ (Patricia, 25 years old, single)

**Subtheme 1.4.3: Ill-treatment:** All participants reported being ill-treated by healthcare providers especially by nurses. They also indicated that if they had a choice they would not return to the public hospitals or clinics. Some participants reported that they would never have any more babies in South Africa because of the ill-treatment they received while attending antenatal clinics, during and after delivery. Many of them, as reported in this study, end up visiting private doctors even though they do not have money to afford their services, but they still feel confident and trust the private sector than the public sector.

‘I was bleeding heavily and so painful but when I called for help, they shouted at me saying I was exaggerating I must keep quiet but I couldn’t, “How can you keep quiet when you feel like dying!” They found things inside I don’t know what you call them placenta or whatever, she (nurse) pushed my tummy until everything came out then pain went down slowly and bleeding stopped.’ (Mercy, 36 years old, married)

#### Sub-theme 1.5: Internalised fear

The findings revealed that ill-treatment resulted in an internalised fear among women refugees. As a result, they are scared to go to the public sector when they are sick or in need of reproductive health services because of previous bad experiences and the fear of facing the very same healthcare providers who mistreated them. Participants said:

‘Because of these experiences I can never have more children in this country.’ (Maria, 43 years old, married)

‘And to be very honest I am very afraid to go to the public hospitals. If I had a choice I will never go back to public hospitals. I don’t wish to go back there.’ (Nadine, 40 years old, married)

#### Sub-theme 1.6: Financial challenges

Participants reported that refugees face financial challenges in the host country making it impossible to access healthcare services in the private sector. The financial challenge was reported in this study as one of the barriers to women refugees in accessing reproductive health services, and as a result they have no choice but to go to the public sector where they feel they are not welcome. Because of financial challenges women refugees are unable to further their education and this leads to unemployment, hence they cannot afford to take care of their basic needs and this makes them more vulnerable and prone to diseases. This is evidenced by the quotation below:

‘We are foreigners here, people are becoming aware of economic challenges and they are accepting family planning as a must because economic challenges are beating them and they are in a situation where they can’t afford to raise many children.’ (Martha, 24 years old, single)

#### Sub-theme 1.7: Shortage of health personnel and overcrowding of public hospitals

Shortages of staff and overcrowding in public institutions are a serious issue facing not only refugees but the citizens as well, and patients are mismanaged while in labour and post-delivery because of shortage of staff, and on many occasions others could not be attended to while seeking reproductive health services including antenatal care because of overcrowding and not enough staff on duty to attend to every patient; they were either turned away or decided to return home untreated after a long period of waiting unsuccessfully. One of the participants made the following statement:

When I was giving birth, a nurse who was attending to me was attending two patients at the same time; when my baby was about to come, she (nurse) was still helping the other lady, so I don’t know if it was a shortage of nurses. As soon as my baby came out the nurse had to rush back to the other lady and I bled until I was gone. It was like my blood was finished, I couldn’t see, I was like blind. (Mercy, 36 years old, married)

#### Sub-theme 1.8: Religious and cultural hegemony

Religious and cultural beliefs were reported to influence women refugees’ health-seeking behaviours. This study revealed that religion and culture play an important role in relation to reproductive health services among women refugees in Durban, KwaZulu-Natal, and their health-seeking behaviours. The following statement was found to be common among all participants:

‘Family planning is not allowed, is considered as a sin. If you’re using it you are a sinner, you are killing babies. But if you use it you hide, you only tell your family. Things like abortion and sex before marriage also not allowed. So, no one talks about family planning when you’re not married. Abortion you can’t even mention it.’ (Amanda, 48 years old, married)

### Theme 2: Positive experiences

#### Sub-theme 2.1: Positive care and treatment

When analysing this theme, it was very clear that the participants had the tendency to compare the care offered in public and the care in private institutions. Some of the participants acknowledged good customer care services in private healthcare institutions, but they are aware of the cost involved as opposed to public institutions where they get free healthcare services. In some rare instances, the participants received good treatment from the nurses or doctors in public hospitals:

‘After that I used family planning for a year then I fell pregnant and I attended the clinic. There my experience was good, the care was good, and they followed me up until I gave birth.’ (Amanda, 48 years old, married).

‘Yaa… After delivery it was okay and I thank God that I met a good doctor who helped and comforted me. Yeah after delivery I didn’t have any problems, they were passing and greeting and ask if I didn’t have any problems.’ (Princess, 47 years old, married)

#### Sub-theme 2.2: Social support

Social support refers to the support, care and assistance from other people. It could be a family support or from friends. In the face of adversity in an unfriendly environment, families’ and friends’ support makes it possible for people who are discriminated against in a foreign country to cope. The participants in this study all mentioned a family member or a friend as someone important to help them bear the pain of the negative experiences they encounter as they seek reproductive health services in public hospitals. This is evidenced by the following statements:

‘My husband was with me and kept on calling them to come.’ (Maria, 43 years old, married)

‘My friend has to raise money for me to go to a private doctor.’ (Mercy, 36 years old, married)

## Discussions

### Theme 1: Negative experiences or challenges

#### Sub-theme 1.1 and 1.2: Medical xenophobia and discrimination

Findings in this study revealed that refugees face discrimination and medical xenophobia when seeking healthcare services at public health facilities in South Africa. This is supported by Crush and Tawodzera (2014) and Zihindula, Meyer-Weitz and Akintola (2015b). Similar results were verified by other researchers, including Nkosi (2014). With specific focus to reproductive health access and utilisation, the existing limited research reports that women have negative experiences with reproductive health services (Krause et al. 2015). These findings were also supported by a study conducted in Botswana, which revealed that women refugees face discrimination, xenophobia, language barriers and other cultural and financial challenges when seeking reproductive health services (Oucho & Ama 2009).

#### Sub-theme 1.3: Language barrier

These findings were supported by Apalata et al. (2007). Their study revealed that women refugees face many challenges with reproductive health services, not only because they cannot communicate but also because there are no interpreters or facilities to convey their messages. As a result, they end up either returning home untreated or have to bring their children or friends who are also not fluent in the local languages to assist with interpretation. Yet, the literature shows that when interpretation is done by an untrained person, there are risks of transmitting a wrong message resulting in wrong diagnosis and treatment that later affect the client negatively (Langlois et al. 2016).

#### Sub-theme 1.4: Unprofessionalism

**Subtheme 1.4.1: Lack of health education and customer care training:** These findings were supported by a study conducted by Ross et al. (2016), which focussed on improving the management and care of refugees in Australian hospitals. Refugees reported negative experiences which they associated with negligence by healthcare professionals, ignorance, lack of education about refugees’ background and very limited customer care training (Dalton-Greyling & Campus 2008), and pointed out that the number of refugees and foreigners entering South Africa is increasing. South Africans will have to be educated concerning refugees and their well-being.

**Subtheme 1.4.2: Failure to obtain consent and lack of confidentiality:** These findings were supported by Apalata et al. (2007), who pointed out the lack of consent and confidentiality, and in their study, women refugees reported being forced to deliver by caesarean without their consent. The issue of confidentiality was found to be a major problem in a refugee camp in Kenya, where women refugees reported that the translators gossiped and disclosed information in the community (Kiura 2012).

**Subtheme 1.4.3: Ill-treatment:** Refugees feel rejected and ill-treated when attending public healthcare institutions. As a result, the majority have decided to no longer attend public hospitals (Apalata et al. 2007).

#### Sub-theme 1.5: Internalised fear

Ill-treatment of the refugees at public health facilities creates an internalised fear that leads women refugees to not seek healthcare services at public health facilities in South Africa (Nkwinika et al. 2014; Zihindula et al. 2016). It was reported that women refugees refused to test for HIV fearing that nurses and counsellors would disclose their status (Zihindula et al. 2016). This explains that not only are women refugees haunted by fear based on previous bad treatment and attitudes from the healthcare providers, but they also do not trust them. The study conducted in India regarding Burmese refugees’ experience accessing healthcare, revealed that ill-treatment played a part in causing internalised fear among refugees who sought reproductive health services at public health facilities (Parmar et al. 2014).

#### Sub-theme 1.6: Financial challenges

These findings were supported by Frenz & Vega (2010) in their study of refugees, which revealed that they face serious financial constraints that do not enable them to afford their basic needs. Refugees are economically disempowered and affording healthcare services is a challenge; as a result they become vulnerable to health-related problems (Zihindula et al. 2015a).

#### Sub-theme 1.7: Shortage of health personnel and overcrowding of public hospitals

It is worth noting that South Africa has long suffered from a shortage of healthcare professionals in its public health sector. A number of reports revealed that the country is currently struggling to fill the gaps of medical doctors, nurses and other healthcare workers, both in rural and urban areas. The shortage of skilled healthcare professionals is not a new phenomenon in the post-apartheid South Africa. Sources indicate that despite the highly reported shortage of skills, the most affected sector remains the National Department of Health (Botha & Rasool 2011; Breier & Erasmus 2009). The problem of staff shortage is further exacerbated by the problems of staff retention and the high turnover rates associated with skills migration that sees a high number of skilled healthcare professionals leaving the country in search of highly paying posts overseas (Theron, Barkhuizen & Du Plessis 2014).

#### Sub-theme 1.8: Religious and cultural hegemony

The findings revealed that both religion and culture in the same way do not accept or support certain services that are a part of reproductive health services. All participants pointed out that family planning and abortion are neither allowed nor supported by their religion and culture. In terms of family planning, they believe that God wants people to multiply as long as they are married. No sex before marriage allowed and abortion is considered as a taboo. Others reported that the use of condoms in a marriage is not something to talk about; it is believed that if a wife wants to use a condom, she is unfaithful, thus hiding something from her husband. Some of the participants also reported that treatment of infertility is not supported by their religion as they believe that God will make things happen in due time. However, because of circumstances in the host country, women refugees found themselves in a position where they have to decide to go against religious and cultural expectations.

Culture prohibits sex before marriage among Assyrian and Karen women refugees living in Australia. It was also discovered that cultural norms affect unmarried women’s access to sexual health services, such as contraception and abortion. The authors concluded that healthcare workers need to be aware of cultural constructions of sex and sexuality, as well as the construction of gendered roles within relationships when offering sexual health services to refugees. Culture and religion were found to be an important factor influencing women’s sexual self-understanding (Ussher et al. 2012). In the study done in a refugee camp in Kenya, Somali women refugees reported that family planning methods are viewed as acts of murder (Kiura 2012).

### Theme 2: Positive experiences

#### Sub-theme 2.1: Positive care and treatment

Irrespective of all negative experiences faced by the participants, the findings revealed some positive care and treatment at some point. Some participants reported positive care and treatment that they received from few employees of the public sectors while utilising reproductive health services.

It was reported that refugees trust the healthcare providers in the private sector more than in the public sector, and this is because of the fact that in private sectors refugees feel like they are treated with respect (Zihindula 2015).

#### Sub-theme 2.2: Social support

Social support is very important in every situation, especially where a vulnerable group of people is concerned – in this case, women refugees. Social support mostly from friends and family plays an important role in times of need in any case. In addition to family members’ and friends’ support, some participants also reported being supported by their fellow patients and in a few cases they received support from healthcare professionals in the public sector.

### Limitations of the study

The study did not involve a bigger sample size. Hence, the findings could not be generalised to the rest of women refugees in South Africa.

This study could have been better if it involved all women refugees from other countries, as this would have provided more information and views on the topic.

## Recommendations

Based on the findings, recommendations were made to the Department of Health regarding the training of healthcare workers in handling refugees’ issues, specifically women in this scenario, in customer care, as well as in the refugee rights to accessing and utilising healthcare services in South Africa. Creating awareness about women refugees’ problems and concerns in relation to reproductive health services will also improve the healthcare providers’ attitudes towards refugees. The Health Department recommended considering either providing an interpreter at facilities most visited by refugees or employing one of the refugees trained and qualified to offer such services, to accommodate refugees in different languages.

## Conclusions

The findings in this study revealed that women refugees continue to face many challenges in their attempt to utilise reproductive health services in public health facilities in Durban, KwaZulu-Natal, South Africa. The study also revealed that women refugees remain the most vulnerable, and their negative experiences and challenges with sexual reproductive health services expose them to a number of health problems mostly related to reproductive health, including complications during pregnancy and post-delivery, which, in turn, becomes a big issue not only for refugees, but also for citizens as well. Furthermore, religion, culture and financial challenges were shown to influence the way women refugees seek reproductive health services.
